# Uncovering the Yeast Diversity in the Female Genital Tract: An Exploration of Spatial Distribution and Antifungal Resistance

**DOI:** 10.3390/pathogens12040595

**Published:** 2023-04-14

**Authors:** Mariana Zagalo Fernandes, Cátia Filipa Caetano, Carlos Gaspar, Ana Sofia Oliveira, Rita Palmeira-de-Oliveira, José Martinez-de-Oliveira, Joana Rolo, Ana Palmeira-de-Oliveira

**Affiliations:** 1 CICS-UBI—Health Sciences Research Centre, University of Beira Interior, 6200-506 Covilhã, Portugal; marianamzf@hotmail.com (M.Z.F.); joanarolo@fcsaude.ubi.pt (J.R.); 2 Faculty of Health Sciences, University of Beira Interior, 6200-506 Covilhã, Portugal; 3 Labfit, Health Products Research and Development Lda, 6200-284 Covilhã, Portugal

**Keywords:** *Candida albicans*, susceptibility, *Rhodotorula* spp., vulvovaginal candidosis

## Abstract

*Candida albicans* is the leading cause of vulvovaginal yeast infections; however, other species are becoming relevant in this niche. The spatial distribution of these fungi in the female genital tract remains poorly understood. In this study, swab samples were collected from 33 patients, first from the anterior vulva and then from the upper third and right lateral wall of the vagina: 16 were with symptoms of vulvovaginal candidiasis and 17 were without characteristic symptoms; furthermore, the genus and species of each isolate were identified. In vitro susceptibility testing for fluconazole and clotrimazole was performed for all isolates. *Candida albicans* was the most common species (63.6%), followed by *Rhodotorula* spp. (51.5%), and then *Candida parapsilosis* (15.2%). *Rhodotorula* spp. and *C. parapsilosis* were more commonly associated with colonization, and *C. albicans* with infection. *Rhodotorula* spp. isolates presented a low susceptibility to fluconazole, with the MIC ranging from 32 to >64 µg/mL. Differences in susceptibility to fluconazole and clotrimazole between the pairs of vaginal and vulvar isolates were found for *Candida albicans*, *Rhodotorula* spp., and *Nakaseomyces glabratus*. The results suggest that different niches may impact the susceptibility profiles of the isolates, as well as their different clinical behaviors.

## 1. Introduction

*Candida* spp. constitute one of the most important genus of opportunistic pathogenic fungi in humans [1,2], comprising the great majority of isolates obtained from fungal invasive and mucosal infections [2,3]. Globally, the five species that belong to the genus *Candida*, presently or formerly, that are more commonly associated with candidosis in humans are *C. albicans* (65.3%), *Nakaseomyces glabratus* (formerly known as *C. glabrata*) (11.3%), *C. tropicalis* (7.2%), *C. parapsilosis* (6.0%), and *Pichia kudriavzevii* (formerly known as *C. krusei*) (2.4%) [4].

Regarding fungal vulvovaginal infections, *C. albicans* is the most prevalent species reported in the majority of studies, accounting for approximately 20.3% to 91.4% of the recovered isolates [5,6]. This applies even when noting that the prevalence of each *Candida* species varies considerably between countries [5,6,7]. Yeasts of the genus *Candida* can asymptomatically colonize the female genital tract and are frequently isolated from this niche. Their excessive proliferation in this niche can lead to inflammation and the development of vulvovaginal candidosis (VVC) [7], which is common among women. In a 2011 surveillance study that was conducted in the United States and in five European countries, 6000 women over 16 years old participated. Depending on the country, 29% to 49% of the participants reported a past VVC diagnosis, while 9% reported a diagnosis of recurrent vulvovaginal candidosis [8]. For symptomatic cases, treatment usually relies on topical or oral azole agents such as clotrimazole and fluconazole, respectively [7].

Some studies suggest an increase in the prevalence of non-*albicans Candida* species as a possible cause of vulvovaginal resistant infections, namely *C. tropicalis*, and *C. parapsilosis*, as well as other species such as *Nakaseomyces glabratus* and *Pichia kudriavzevii* [5,6,9]. Some of these species, such as *N. glabratus* and *P. kudriavzevii*, have also shown a lower susceptibility than *C. albicans* to the azole agents used in the treatment of vaginal candidosis [2,9,10].

New fungal species, such as those belonging to the *Rhodotorula* genus, have also been emerging as potential pathogens of the female genital tract. Isolates of this genus are commonly found in the genital samples of either asymptomatic or symptomatic women, but they are also more frequently associated with asymptomatic cases [11,12]. The most common species of this genus isolated from female genital samples is *R. mucilaginosa*, which is also known as *R. rubra* [11,13,14]. This fungus is considered ubiquitous and saprophytic; it is found in different ecosystems, in foods and beverages, and in some mammals and birds [15].

The vagina and vulva are quite different environments, in terms of humidity, temperature, acidity, carbon sources, nutrients and inhibitors, microbial communities, their contact and exchange with the external environment, and in their immune responses, as well as among other factors that can directly affect the microbiota present in their ecosystems [16].

Little information currently exists on the spatial distribution of the yeast species in the female genital tract, either in the case of infection or in the case of asymptomatic colonization. In this study, we aimed to characterize the spatial distribution and susceptibility of different yeast species—which were collected from the vaginal and vulvar samples of women with different clinical states—to fluconazole and clotrimazole in order to clarify if there is any influence from the recovery niche in the susceptibility profile to the most commonly used antifungals, as well as whether their temporal appearance was in accordance with the clinical evolution.

## 2. Materials and Methods

### 2.1. Sample Collection, Specimen Isolation, and Clinical Status

Swab samples were collected from the vulva and vagina of the patients at specialist appointments (one sample from each anatomical site per patient per appointment). The first cotton swab sampled the anterior interlabial sulci at both sides, while a second swab collected the fluid along the right vaginal wall up and down, and without touching the vulva. All patients were of childbearing age with a present infection or with a history of infection. These samples were collected by one gynecologist during 61 consultations with 33 patients between October 2019 and August 2022. After collection, the samples were separately seeded in Petri dishes within a Sabouraud Dextrose Agar (SDA, VWR, Radnor, PA, USA) medium and were duly catalogued according to clinical history. 

Each colony with a distinct morphology in each plate was isolated and identified, as is mentioned below in the “Species identification” section. Specimens from pink colonies were incubated for 36–48 h at 25 °C, and all other isolates were incubated for 24–48 h at 37 °C. All isolates were stored frozen in cryogenic vials within a brain heart infusion (BHI, VWR, Radnor, PA, USA) broth and in 20% glycerol at −80 °C until needed for further analysis.

Patients were not recruited or selected for inclusion in this study; the samples constituted convenience samples as they were collected for diagnostic purposes only, during or following vulvovaginal clinical episodes, either recent or remote ones. The study was approved by the Ethics Committee of the University of Beira Interior (CE-UBI-Pj-2018-022).

Information on clinical status was provided for all 33 patients. The classification assigned depended on the symptoms presented, with the following criteria being considered relevant for the diagnosis of infection: thick curdy vaginal discharge, burning sensation, itching, inflammation, irritation and/or cracking on the vagina or vulva, as well as pain during sexual intercourse. The patients that did not show these symptoms at the time of sampling were considered asymptomatic.

### 2.2. Species Identification

After thawing, each isolate was plated on SDA. Isolates from pink colonies were incubated for 36–48 h at 25 °C, and all remaining isolates were incubated for 24–48 h at 37 °C. 

The *C. albicans* isolates were identified by two subcultures in a chromogenic medium, i.e., chromID TM Candida Agar (CAN2, BioMérieux, Marcy-l’Étoile, France). The genus *Rhodotorula* was identified via macroscopic and microscopic analyses of its characteristic colonies on SDA. The remaining isolates´ species identification was performed by an automated analysis of their biochemical profiles using Vitek^®^ (BioMérieux, Marcy-l’Étoile, France). Vitek^®^ does not allow the differentiation between *Rhodotorula glutinis* and *Rhodotorula mucilaginosa*, and the macroscopic and microscopic observation also does not allow one to discern between the species of *Rhodotorula*; therefore, we only identified this group of isolates up to the genus. The species nomenclature was performed by following the recommendations on https://www.ncbi.nlm.nih.gov/Taxonomy/Browser/wwwtax.cgi, accessed on 10 April 2023. 

### 2.3. Species Prevalence Comparison with Some Existing Studies 

The search engines PubMed, Google Scholar, Scopus, and Web of Science were used to find relevant scientific articles that characterized the prevalence of different species of yeast from the genital samples of symptomatic or asymptomatic women. The terms “*Candida*/*Rhodotorula*/yeast prevalence in vagina/genital” were used. Preference was given to studies from Europe. The data were standardized to represent the proportion of each species in the total sample from asymptomatic or symptomatic patients in each study and are presented in the form of a table.

### 2.4. Susceptibility Testing by Broth Microdilution

In vitro yeast susceptibility testing for fluconazole (Thermo Fisher Scientific, Waltham, MA, USA) and clotrimazole (Sigma-Aldrich, St. Louis, MO, USA) was performed using 96-well microplates, in accordance with the guidelines detailed by the European Committee on Antimicrobial Susceptibility Testing (EUCAST, 2020). 

For the *Rhodotorula* spp. isolates, fluconazole and clotrimazole suspensions were prepared in a Yeast Extract-Peptone-Dextrose (YPD, Fisher BioReagents, Waltham, MA, USA) broth medium. They were placed in the wells in successive half-dilutions, such that the concentrations ranged from 2 to 64 µg/mL. For the inoculum preparation, the isolates were plated on SDA and incubated for 36–48 h at 25 °C. After incubation, each isolate was suspended in 5 mL of NaCl at a 0.85% to an optical density (OD) of 0.5 McFarland, which corresponds to approximately 1–5 × 10^6^ CFU/mL. The suspension was then diluted to 1:1000 in YPD broth medium and then further diluted to 1:2 when applied to the plate wells in order to achieve approximately 0.5 × 10^3^ to 2.5 × 10^3^ CFU/mL. The microplates were incubated for 48 h at 25 °C. Optical density readings were taken at 24 h and 48 h using the xMark^TM^ Microplate Absorbance Spectrophotometer (BIO RAD, Hercules, CA, USA) at an absorbance of 600 nm.

For the remaining isolates, fluconazole and clotrimazole suspensions were prepared in a Roswell Park Memorial Institute (RPMI-1640, Sigma-Aldrich, St. Louis, MO, USA) broth medium and placed in the wells in successive half-dilutions, such that the concentrations ranged from 2 to 64 µg/mL. The inoculums were prepared by plating the isolates on SDA and were then incubated for 24 h at 37 °C. Each isolate was suspended in 5 mL of NaCl at a 0.85% to an optical density (OD) of 0.5 McFarland, which corresponds to approximately 1–5 × 10^6^ CFU/mL. The suspension was diluted to 1:1000 in an RMPI broth medium and then further diluted to 1:2, when applied to the plate wells, to approximately 0.5 × 10^3^ to 2.5 × 10^3^ CFU/mL. The microplates were incubated for 48 h at 37 °C. Optical density readings were taken at 24 and 48 h using a spectrophotometer at an absorbance of 600 nm.

### 2.5. Statistical Analyses

The data in this study were statistically analyzed using the two-tailed t-student test. The groups compared were the isolates recovered from patients with characteristic symptoms of vulvovaginal candidosis against the isolates recovered from patients without characteristic symptoms, and the isolates recovered from the vulva against isolates recovered from the vagina. For both comparisons, the null hypothesis considered was that there was no significant difference in the susceptibility to fluconazole or clotrimazole between the two groups of isolates; this was achieved by comparing the minimum inhibitory concentration results. We used a significance level of *p* < 0.05 to determine statistical significance. The data were analyzed using RStudio (2022), assuming equal variances between the two groups. We interpreted the results by comparing the calculated t-value to the critical t-value at the specified significance level, and we considered the result statistically significant if the *p*-value was less than 0.05. 

## 3. Results

### 3.1. Sample Characterization

Among the 33 patients, 12 were asymptomatic at the time of sampling. Twelve other patients were symptomatic at the time of sampling, as per the following: vulvar infection (2), vulvovaginal infection (3), vaginal infection (4), and uncharacteristic symptoms (3). The remaining nine patients had episodes of infection and asymptomatic episodes, in which yeast isolates were collected. These data are provided in greater detail in Table 1.

### 3.2. Species Identification

A total of 102 isolates were obtained from the 122 samples that were collected from the 33 patients (one sample from each anatomical site per patient per appointment). The detailed information regarding the isolates’ origin and their clinical context is presented in Table 1. 

**Figure 1:**
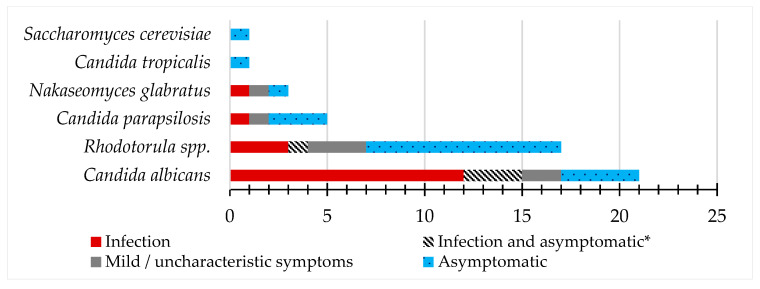
Species isolated from vaginal and vulval samples according to the clinical state information. Each species was considered one time per patient, even if the same species appears in more than one appointment. *—Infection was followed by an asymptomatic diagnosis after treatment.

The most prevalent species detected was *C. albicans* in 63.6% (21/33) of patients, followed by *Rhodotorula* spp. in 51.5% (17/33), *C. parapsilosis* in 15.2% (5/33), *N. glabratus* in 9.1% (3/33), *C. tropicalis* in 3.0% (1/33), and *Saccharomyces cerevisiae* in 3.0% (1/33). According to the clinical state, *C. albicans* was retrieved more frequently from infection cases, while *Rhodotorula spp*. and *C. parapsilosis* were mainly retrieved from asymptomatic cases, as is demonstrated in Figure 1. 

Although in 77.0% (47/61) of appointments only one species was isolated from both the vaginal and vulvar samples per patient, two species were isolated from the same patient in 14 cases; namely, seven combinations of *C. albicans* and *Rhodotorula* spp., one of *C. albicans* and *C. parapsilosis*, two of *C. parapsilosis* and *Rhodotorula* spp., three of *N. glabratus* and *Rhodotorula* spp., and one of *S. cerevisiae* and *Rhodotorula* spp., as is described in Table 2.

### 3.3. Species Prevalence Comparison with Some Existing Studies 

The ratio between the yeast species that were isolated from female genital samples in this study and in seven other different studies is presented in Table 3. The prevalence of each species varied considerably between studies, even in studies that were carried out in the same country, as is the case with two studies in Portugal and two studies in Italy. *C. albicans* and *N. glabratus* were the most prevalent species in most studies, and—among the symptomatic cases—*C. albicans* was the most common species across all studies, ranging from 42.9% to 89.3% of the isolated species. In the four studies that presented symptomatic and asymptomatic cases, *C. albicans* prevalence was lower in the asymptomatic cases. The studies varied in their inclusion criteria, in aspects such as pregnancy and the use of antifungal agents in the 15 days prior to sampling, which could affect the prevalence of the yeast species.

### 3.4. Spatial Distribution of the Isolates

**Figure 2:**
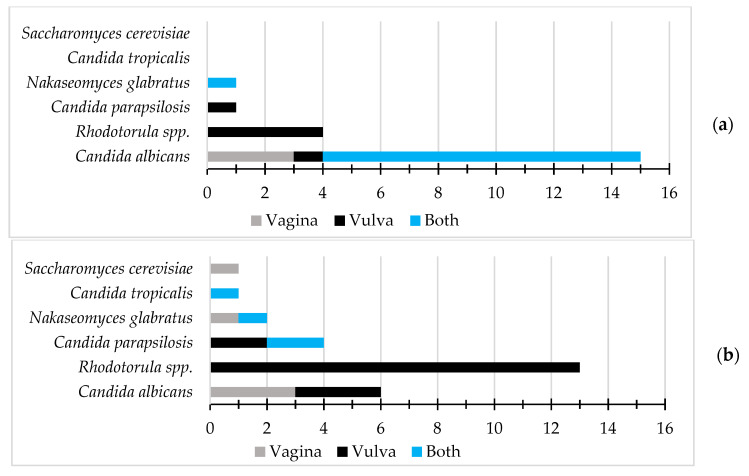
Isolate spatial distribution: (**a**) among the samples recovered from patients presenting characteristic symptoms of yeast genital infection; (**b**) among the samples recovered from patients without symptoms that are characteristic of yeast genital infection. Each species was considered one time per patient, even if the same species appeared in more than one appointment.

*Rhodotorula* spp. isolates were only obtained from vulvar samples. *C. albicans* isolates were slightly more prevalent in the vagina than in the vulva, in both infection and non-infection cases. In infection cases, *C. albicans* was isolated from both anatomical sites in 73.3% (11/15) of cases, only from the vagina in 20.0% (3/15), and only from the vulva in 6.7% (1/15). In non-infection cases, it was retrieved only from the vagina or vulva in half of the cases each. The spatial distribution of the isolates according to the clinical state of the patients is represented in Figure 2. 

### 3.5. Antifungal Susceptibility 

All 102 isolates obtained in this study via the broth microdilution method were subjected to fluconazole and clotrimazole susceptibility tests. These isolates included 45 *C. albicans*, 15 *N. glabratus*, 4 *C. tropicalis*, 7 *C. parapsilosis*, 1 *S. cerevisiae*, and 30 *Rhodotorula* spp. 

Considering the EUCAST breakpoints for fluconazole (susceptible ≤ 2 µg/mL, resistant > 4 µg/mL for *C. albicans* and susceptible ≤ 0.0014 µg/mL, resistant > 16 µg/mL for *N. glabratus*), 8.9% (4/45) of *C. albicans* isolates were considered resistant and the remaining 91.1% (41/45) were considered susceptible. For *N. glabratus*, 53.3% (8/15) of the isolates were considered susceptible and 46.7% (7/15) were considered resistant. All isolates of *C. tropicalis* and *C. parapsilosis* were considered susceptible. No breakpoints for fluconazole are currently set for *S. cerevisiae*, nor for *Rhodotorula* spp. There are also no established breakpoints for clotrimazole.

The minimum inhibitory concentration (MIC) of fluconazole required to inhibit 90% of *C. albicans* isolates was ≤2 µg/mL; for *N. glabratus* isolates, it was >64 µg/mL; and for *Rhodotorula* spp. isolates, it was also >64 µg/mL. All the isolates from the remaining species had a MIC ≤ 2 µg/mL. 

Among the *C. albicans* isolates retrieved from distinct clinical statuses, 10.5% (4/38) of those from patients with characteristic symptoms of vulvovaginal candidosis had a fluconazole MIC > 4 µg/mL, while none of those from patients without symptoms had a MIC > 2 µg/mL (0/7). Of the *N. glabratus* isolates, 88.9% (8/9) from one patient with characteristic symptoms had a fluconazole MIC > 4 µg/mL, while none (0/9) from two patients without symptoms had a MIC > 4 µg/mL; furthermore, the MIC difference was significant (*p*-value = 0.0013). For *Rhodotorula* spp., 100% (15/15) of isolates from patients with and without characteristic symptoms had a fluconazole MIC > 4 µg/mL. These results are presented in Table 4.

The MIC of clotrimazole required to inhibit 90% of *C. albicans* isolates was ≤2 µg/mL; for *N. glabratus* isolates, it was 4 µg/mL; and for *Rhodotorula* spp. isolates, it was 64 µg/mL. All the isolates from the remaining species had a MIC ≤ 2 µg/mL.

Among the *C. albicans* isolates from patients with characteristic symptoms of vulvovaginal candidosis, 2.6% (11/38) had a clotrimazole MIC > 4 µg/mL, while none from patients without characteristic symptoms had a MIC > 2 µg/mL (0/7). Of the *N. glabratus* isolates, 50.0% (3/6) from one patient with characteristic symptoms had a clotrimazole MIC > 4 µg/mL, while 22.2% (2/9) from two patients without symptoms had a MIC > 4 µg/mL. For *Rhodotorula* spp., 60.0% (9/15) of isolates from patients with characteristic symptoms had a clotrimazole MIC > 4 µg/mL, while none (0/15) from patients without characteristic symptoms had a MIC > 2 µg/mL; furthermore, the MIC difference was significant (*p*-value = 0.003).

For all remaining cases, there was no statistically significant difference in fluconazole or clotrimazole susceptibility profiles.

Based on the sample site, *C. albicans* and *N. glabratus* isolates from the vulva showed a higher susceptibility to both fluconazole and clotrimazole (Table 5). This trend was also observed when comparing sample pairs. Among the 24 cases where isolates of the same species were isolated from both the vaginal and vulvar samples, in 20.8% (5/24) of the pairs, the vulvar isolates were less susceptible to fluconazole and/or clotrimazole, and in 4.2% (1/24) of the pairs, the vaginal isolates were less susceptible, as is shown in Table 6. The observed differences were not statistically significant (*p*-value > 0.05).

## 4. Discussion

The vulva and vagina, although close, are two very different environments for microorganisms [16]. *Candida* spp. can cause infections both in the vulva (vulvitis) and in the vagina (vaginitis). Vulvitis and vaginitis that are caused by *Candida* spp. can occur separately or together, but in both cases it is usually referred to as vulvovaginal candidosis [7,23,24]. 

This disease is common and affects women worldwide [5,7]; however, little information is currently available on the spatial distribution and characteristics of the yeasts isolated from these two niches, both in the case of infection and colonization. Therefore, this study was carried out to determine the prevalence of the different species isolated from the vulva and the vagina, which were collected from a group of women with a present or past history of VVC. We also aimed to determine the susceptibility profiles of these isolates to fluconazole and clotrimazole, as well as their anatomical distribution in the female genital region in asymptomatic women or in patients with symptoms and signs of genital fungal infection.

In this study, we obtained isolates of *C. albicans*, *Rhodotorula* spp., *C. parapsilosis*, *N. glabratus*, *C. tropicalis*, and *Saccharomyces cerevisiae*. All these species have already been reported several times in samples from the female genital region, particularly from the vagina [9,11,12,17,18,19,20,21,22,25]. We analyzed the samples from patients with symptoms of vaginal, vulvovaginal, or vulvar infections, as well as from patients with uncharacteristic or mild symptoms and asymptomatic patients. The frequency of yeast species isolated varied among these groups. Similarly, other studies that analyzed yeast isolates from vaginal samples of both infection and asymptomatic cases also detected differences in the proportions of the species obtained, as can be seen in four of the studies presented in Table 3. Considering that in chronic cases of vulvovaginal candidosis investigators reported that, in approximately 53% of these cases, the episodes were due to genetically related isolates [26], we decided to count each species only once per patient per anatomical site in order to avoid characterizing the same isolate that could have survived between infections.

In the studies we used for comparison, as is represented in Table 3, the *Candida* species most frequently isolated from cases of infection was *C. albicans*, followed by *N. glabratus* [17,18,19,20,21] or *C. parapsilosis* [11,22], and the same is true for the asymptomatic cases, although a higher proportion of *C. parapsilosis* and a greater diversity of species were found [11,17,20]. In this study, the *Candida* species obtained from vulvar, vaginal, and vulvovaginal infections were also *C. albicans*, *N. glabratus*, and *C. parapsilosis*. The most evident difference in our study, particularly when compared to the other Portuguese study mentioned, is the absence of isolates of the species *Meyerozyma guilliermondii* (formerly known as *C. guilliermondii*), *Clavispora lusitaniae* (formerly known as *Candida lusitaniae*), *Nakaseomyces nivariensis* (formerly known as *Candida nivariensis*), and *P. kudriavzevii*. We thought of two possible reasons for this, one being the small size of our sample, and the other being the distance and different characteristics of the study populations being enough to observe slight epidemiological differences [17].

Accounting for all species, *Rhodotorula* spp. was the second most prevalent in the infection cases. However, we should mention that in all cases of infection, other species besides *Rhodotorula* spp. were present, which can be observed in Table 1. In the literature, we did not find any report on *Rhodotorula* spp. causing genital infection and we were also unable to associate *Rhodotorula* spp. as an isolate causative agent of infection, pointing to a possible non-pathogenic behavior in these niches. It is also important to note that *R. mucilaginosa,* the *Rhodotorula* species most frequently isolated from female genital samples, has already been linked to multiple cases of fungemia and other infections in other anatomical sites in vulnerable patients [15].

Among the samples from patients without symptoms that are characteristic of yeast genital infection, such as the asymptomatic cases of the mentioned studies, we also observed a greater diversity of species, namely *C. tropicalis* and *S. cerevisiae*, and a higher proportion of *C. parapsilosis* and *Rhodotorula* spp. Unlike *Rhodotorula* spp., *C. parapsilosis* isolates have already been associated with symptoms of vulvovaginal infection [21].

Similar to other studies that analyzed yeast isolates from genital samples of symptomatic and asymptomatic patients, we also detected the presence of more than one species per sample. In 12 of the 13 cases, we identified *Rhodotorula* spp. as one of the species and we only obtained one combination of different *Candida* species: *C. albicans* and *C. parapsilosis*. However, it is important to note that, macroscopically, the colonies of different *Candida* species appear identical in different culture media [27], such that the lack of distinction between these isolates in the samples can lead to an underestimation of co-colonization events.

Our results also showed a clear pattern in the spatial distribution of the yeast isolates. All species found in more than one sample were present in both vaginal and vulvar samples, except for *Rhodotorula* spp., which was only isolated from vulvar samples. We did not find any studies in the literature that distinguished the presence of yeast isolates in the vulva from their presence in the vagina either from asymptomatic or symptomatic patients. Therefore, to our knowledge, the possible preference of *Rhodotorula* spp. isolates for the vulva that we observed is described here for the first time. 

We observed that *C. parapsilosis* isolates were more frequently obtained from vulvar samples, while *N. glabratus* isolates were more frequently isolated from vaginal samples; however, due to the lack of bibliographic material for comparison and the small size of our sample, we cannot speculate whether these results may be due to chance. Future studies with a larger number of samples may clarify this observation.

In the context of infection, most *C. albicans* isolates were obtained from samples from both anatomical sites simultaneously; however, in the absence of the characteristic symptoms of infection, isolates were obtained only from the vagina or vulva. In both contexts, infection or absence of characteristic symptoms of infection, the presence of *C. albicans* isolates in the vagina was higher than those found in the vulva. As already mentioned, we did not find this type of analysis in the literature. As such, we were unable to compare it with previous results. However, as is common in the context of infection, high proliferation of *C. albicans* occurs [28], which leads us to suggest the hypothesis that the risk of contagion from both anatomical sites may be higher. This is determined by mainly taking into account the proximity of the vulva and vagina, as well as the presence of vaginal discharges in the vulva, with *C. albicans* isolates.

The characterization of the susceptibility profiles of both clinical and colonization isolates to antifungal agents is important for epidemiological surveillance, especially given the increase in reports of isolates that exhibit inherent or developed resistance to antifungal drugs [29]. Azole agents such as fluconazole are fungistatic and not fungicidal; therefore, the appearance of resistant isolates in the population exposed to this agent is more common [30]. Some of the tested isolates came from patients who had already undergone previous rounds of treatment with azole agents; as such, the existence of some resistant isolates would be expected.

In our study, we found that 8.9% of *C. albicans* isolates were resistant to fluconazole according to EUCAST breakpoints, and 2.2% had decreased susceptibility to clotrimazole. The proportion of isolates with low susceptibility to both antifungals was higher among the isolates collected from patients with characteristic symptoms of vulvovaginal candidosis when compared to those without these symptoms, although the difference was not statistically significant. Regarding clotrimazole susceptibility, this trend was also observed with the only isolate with a clotrimazole MIC being higher than 4 µg/mL belonging to a patient with characteristic symptoms of vulvovaginal candidosis. In the studies already mentioned, with respect to the analyzed yeast genital isolates from asymptomatic and symptomatic patients, none differentiated the susceptibility results regarding the clinical status of the patients. In a study from Italy in 1997, which performed susceptibility testing in the *Candida* species to multiple antifungals including fluconazole, no significant difference between the two groups of *C. albicans* isolates was found, although this was in a small sample size (the symptomatic group comprised 6 isolates and the carrier group of 13 isolates) [31]. The small sample size of both studies may have influenced the statistical results. As such, future studies with a larger number of isolates may shed light on the pattern we observe; however, we hypothesize that the higher prevalence of resistant isolates may be due to the use of the respective antifungals in the treatments performed previously, and due to the development of resistance or the selection of resistant isolates.

For the *N. glabratus* isolates, we observed that 46.7% showed resistance to fluconazole according to EUCAST breakpoints, and more than half had decreased susceptibility to clotrimazole. The fluconazole susceptibility of the isolates from one asymptomatic woman was significantly lower than the isolates from patients with symptoms. We consider this result with great caution because we are aware that the isolates may be very similar genetically due to their shared origin in the symptomatic group. The resistance of a high proportion of *N. glabratus* isolates to fluconazole is already well known in the literature and has shown a slight tendency to increase over the years [32], having been associated with overexpression of efflux pumps [33]. Although not abnormal, our results could be considered higher when compared to those of previous studies conducted in Portugal [17]. The isolates of the species that we tested were collected from only three different patients; as such, our results may be influenced by the low genetic diversity among the isolates.

Interestingly, we observed that the vulvar isolates of *N. glabratus* and *C. albicans* demonstrated a greater susceptibility to, at least, one of the antifungals tested when compared to the vaginal isolates. This result may be due to several factors, such as the possible lower concentration of fluconazole that was experienced by vulvar isolates in previous rounds of treatment due to pharmacokinetics, leading to different evolutionary pressure, epigenetic alterations, or strain substitution [30,34]. If significant, these differences could, in some cases, make the vulva a reservoir between episodes of infection. This observation was not possible for any other species because all isolates of *C. tropicalis, C. parapsilosis*, and *S. cerevisiae* presented a MIC to clotrimazole and fluconazole that was equal or lower than 2 µg/mL. Moreover, all *Rhodotorula* spp. isolates were recovered from the vulvar samples.

## 5. Conclusions

The presence and proportion of certain yeast species appeared to vary according to the clinical condition considered. Namely, the isolates of *Rhodotorula* spp. and *C. parapsilosis* appeared to be more associated with colonization, whereas *C. albicans* isolates were more often associated with infection. Differences in susceptibility to azoles were observed between vaginal and vulvar isolates, indicating that the different selective pressures of these two niches may be shaping the susceptibility profiles of the isolates differently.

In summary, the present study points to the importance and need to further explore the impact of the environment in the establishment of fungal colonization and infection in the lower female genital tract.

## Figures and Tables

**Table 1:** Patient ID, clinical status, sample site, and the species of the isolates obtained.

ID	Clinical Status	Sample Site	Isolated Species	ID	Clinical Status	Sample Site	Isolated Species
1	Vulvar infection	Vagina	*C. albicans*	13	Vaginal infection	Vagina	*C. albicans*
		Vulva	*C. parapsilosis*			Vulva	*C. albicans*
2	Vulvovaginal infection	Vagina	*N. glabratus*	14	Uncharacteristic symptoms	Vagina	*C. parapsilosis*
		Vulva	*N. glabratus*			Vulva	*C. parapsilosis; Rhodotorula* spp.
	Vulvovaginal infection	Vagina	*N. glabratus*	15	Asymptomatic	Vulva	*C. parapsilosis*
		Vulva	*N. glabratus*	16	Asymptomatic	Vulva	*Rhodotorula* spp.
	Vulvovaginal infection	Vagina	*N. glabratus*			Vagina	*S. cerevisiae*
		Vulva	*N. glabratus*	17	Asymptomatic	Vagina	*N. glabratus*
3	Asymptomatic	Vagina	*C. tropicalis*			Vulva	*Rhodotorula* spp.
		Vulva	*C. tropicalis*		Asymptomatic	Vulva	*Rhodotorula* spp.
	Asymptomatic	Vagina	*C. tropicalis*	18	Vaginal infection	Vagina	*C. albicans*
		Vulva	*C. tropicalis*	19	Asymptomatic	Vulva	*Rhodotorula* spp.
4	Vulvovaginal infection	Vagina	*C. albicans*	20	Vulvar infection	Vagina	*C. albicans*
		Vulva	*C. albicans*			Vulva	*C. albicans*
	Asymptomatic	Vulva	*C. albicans*		Asymptomatic	Vulva	*Rhodotorula* spp.
	Vulvovaginal infection	Vagina	*C. albicans*	21	Vaginal infection	Vagina	*C. albicans*
		Vulva	*C. albicans*			Vulva	*C. albicans*
5	Vulvar infection	Vulva	*C. albicans*	22	Asymptomatic	Vagina	*C. parapsilosis*
	Asymptomatic	Vulva	*C. albicans*			Vulva	*C. parapsilosis; Rhodotorula* spp.
	Vulvar infection	Vulva	*C. albicans*	23	Uncharacteristic symptoms	Vagina	*C. albicans*
	Asymptomatic	Vulva	*C. albicans*	24	Asymptomatic	Vulva	*Rhodotorula* spp.
	Vulvar infection	Vagina	*C. albicans*	25	Asymptomatic	Vulva	*Rhodotorula* spp.
		Vulva	*C. albicans*	26	Asymptomatic	Vulva	*Rhodotorula* spp.
	Vulvar infection	Vulva	*C. albicans*	27	Vaginal infection	Vagina	*C. albicans*
6	Uncharacteristic symptoms	Vulva	*Rhodotorula* spp.			Vulva	*Rhodotorula* spp.
	Asymptomatic	Vulva	*C. parapsilosis*		Asymptomatic	Vulva	*Rhodotorula* spp.
7	Mild symptoms	Vagina	*N. glabratus*		Asymptomatic	Vulva	*Rhodotorula* spp.
		Vulva	*N. glabratus; Rhodotorula* spp.		Vulvovaginal infection	Vagina	*C. albicans*
	Mild symptoms	Vagina	*N. glabratus*			Vulva	*C. albicans; Rhodotorula* spp.
		Vulva	*N. glabratus*		Asymptomatic	Vulva	*Rhodotorula* spp.
	Mild symptoms	Vagina	*N. glabratus*		Asymptomatic	Vagina	*C. albicans*
		Vulva	*N. glabratus*			Vulva	*Rhodotorula* spp.
	Mild symptoms	Vagina	*N. glabratus*	28	Asymptomatic	Vagina	*C. albicans*
		Vulva	*N. glabratus; Rhodotorula* spp.		Asymptomatic	Vulva	Unidentified
	Asymptomatic	Vagina	*C. albicans*	29	Vulvovaginal infection	Vagina	*C. albicans*
8	Asymptomatic	Vulva	*C. albicans*			Vulva	*C. albicans; Rhodotorula* spp.
9	Vulvovaginal infection	Vagina	*C. albicans*		Asymptomatic	Vagina	Unidentified
		Vulva	*C. albicans*	30	Uncharacteristic symptoms	Vulva	*C. albicans*
10	Vaginal infection	Vagina	*C. albicans*		Uncharacteristic symptoms	Vulva	*C. albicans*
11	Asymptomatic	Vulva	*C. albicans; Rhodotorula* spp.	31	Vulvovaginal infection	Vagina	*C. albicans*
12	Vulvovaginal infection	Vagina	*C. albicans*			Vulva	*C. albicans*
		Vulva	*C. albicans; Rhodotorula* spp.		Asymptomatic	Vulva	*Rhodotorula* spp.
	Asymptomatic	Vulva	*Rhodotorula* spp.	32	Vulvar infection	Vulva	*C. albicans; Rhodotorula* spp.
	Vulvovaginal infection	Vagina	*C. albicans*	33	Vulvovaginal infection	Vagina	*C. albicans*
		Vulva	*C. albicans*			Vulva	*C. albicans*

**Table 2:** Cases where more than one species was isolated from one patient per consultation, with patient ID, clinical status, sample site, and the species described.

ID	Clinical Status	Sample Site	Isolated Species
1	Vulvar infection	Vagina	*C. albicans*
		Vulva	*C. parapsilosis*
7	Mild symptoms	Vagina	*N. glabratus*
		Vulva	*N. glabratus; Rhodotorula* spp.
	Mild symptoms	Vagina	*N. glabratus*
		Vulva	*N. glabratus; Rhodotorula* spp.
11	Asymptomatic	Vulva	*C. albicans; Rhodotorula* spp.
12	Vulvovaginal infection	Vagina	*C. albicans*
		Vulva	*C. albicans; Rhodotorula* spp.
14	Uncharacteristic symptoms	Vagina	*C. parapsilosis*
		Vulva	*C. parapsilosis; Rhodotorula* spp.
16	Asymptomatic	Vagina	*Saccharomyces cerevisiae*
		Vulva	*Rhodotorula* spp.
17	Asymptomatic	Vagina	*N. glabratus*
		Vulva	*Rhodotorula* spp.
22	Asymptomatic	Vagina	*C. parapsilosis*
		Vulva	*C. parapsilosis; Rhodotorula* spp.
27	Vaginal infection	Vagina	*C. albicans*
		Vulva	*Rhodotorula* spp.
	Vulvovaginal infection	Vagina	*C. albicans*
		Vulva	*C. albicans; Rhodotorula* spp.
	Asymptomatic	Vagina	*C. albicans*
		Vulva	*Rhodotorula* spp.
29	Vulvovaginal infection	Vagina	*C. albicans*
		Vulva	*C. albicans; Rhodotorula* spp.
32	Vulvar infection	Vulva	*C. albicans; Rhodotorula* spp.

**Table 3:** Ratio between the different yeast species that were isolated from the vulvovaginal samples obtained from infected and asymptomatic patients, in different studies.

Clinical Status	Total	CA	NG	CT	CP	MG	PK	CL	NN	Other *Candida* spp.	*R.* spp.	SC	Country and Ref.
Symptomatic	21	71.4%	4.8%	0%	4.8%	0%	0%	0%	0%	0%	19.0%	0%	Portugal, this study
	93	69.7%	19.4%	0%	5.4%	1.1%	1.1%	2.2%	1.1%	-	-	-	Portugal [17]
	3332	42.9%	36.2%	12.1%	-	-	8.7%	-	-	-	-	-	Italy [18]
	898	78.1%	14.8%	2.3%	0.6%	-	4.0%	-	-	0.2%	-	-	Italy [19]
	28	89.3%	10.7%	0%	0%	0%	0%	-	-	-	-	-	Iran [20]
	103	55.3%	8.7%	6.8%	11.7%	2.9%	1.0%	1.0%	-	11.6%	1.0%	-	Brazil [11]
	635	72.0%	11.7%	-	8.5%	-	-	0.8%	-	-	2.8%	1.4%	USA [21]
	255	51.4%	11.4%	4.3%	25.9%	-	3.9%	0.4%	0.4%	1.6%	-	0.8%	Vietnam [22]
Asymptomatic	27	22.2%	7.4%	3.7%	14.8%	0%	0%	0%	0%	0%	48.1%	3.7%	Portugal, this study
	275	57.1%	21.8%	1.1%	11.3%	4.0%	0.7%	1.8%	2.2%	-	-	-	Portugal [17]
	255	51.4%	11.4%	4.3%	25.9%	-	3.9%	0.4%	0.4%	1.6%	-	0.8%	Iran [20]
	54	44.4%	20.4%	3.7%	18.5%	0%	1.9%	0%	-	5.6%	5.6%	-	Brazil [11]

Abbreviations: CA, *Candida albicans*; NG, *Nakaseomyces glabratus*; CT, *Candida tropicalis*; CP, *Candida parapsilosis*; MG, *Meyerozyma guilliermondii*; PK, *Pichia kudriavzevii*; CL, *Candida lusitaniae*; NN, *Nakaseomyces nivariensis*; SC, *Saccharomyces cerevisiae*. *R.* spp., *Rhodotorula* spp.

**Table 4:** The susceptibility profiles of *Candida* and *Rhodotorula* isolates in patients with and without vulvovaginal candidosis characteristic symptoms to fluconazole (FLU) and clotrimazole (CLOT).

Species	Patient with Vulvovaginal Candidosis Characteristic Symptoms	No. of Patients	No. of Isolates	% of Isolates with FLU MIC > 4 µg/mL	% of Isolates with CLOT MIC > 4 µg/mL
*C. albicans*	Yes	15	38	10.5%	2.6%
	No	6	7	0%	0%
*N. glabratus*	Yes	1	6	0%	50.0%
	No	2	9	88.9%	22.2%
*Rhodotorula* spp.	Yes	4	15	100%	60.0%
	No	13	15	100%	0%

**Table 5:** The minimum inhibitory concentrations (MIC) of all isolates from the vagina and vulva to fluconazole (FLU) and clotrimazole (CLOT), as determined by broth microdilution.

Antifungal	Species	Site	No. of Isolates	MIC Range (µg/mL)	≤2	2–4	4–8	8–16	16–32	32–64	>64
FLU	*C. albicans*	Vagina	21	≤2–>64	90.5%	0%	0%	0%	0%	4.8%	4.8%
		Vulva	24	≤2–64	91.7%	0%	4.2%	0%	0%	4.2%	0%
	*N. glabratus*	Vagina	8	≤2–>64	37.5%	12.5%	0%	12.5%	0%	12.5%	25.0%
		Vulva	7	≤2–64	42.9%	0%	0%	0%	42.9%	14.3%	0%
	*C. tropicalis*	Vagina	2	≤2	100.0%	0%	0%	0%	0%	0%	0%
		Vulva	2	≤2	100.0%	0%	0%	0%	0%	0%	0%
	*C. parapsilosis*	Vagina	2	≤2	100.0%	0%	0%	0%	0%	0%	0%
		Vulva	5	≤2	100.0%	0%	0%	0%	0%	0%	0%
	*S. cerevisiae*	Vagina	1	≤2	100.0%	0%	0%	0%	0%	0%	0%
		Vulva	0	-	0%	0%	0%	0%	0%	0%	0%
	*Rhodotorula* spp.	Vagina	0	-	0%	0%	0%	0%	0%	0%	0%
		Vulva	30	32–>64	0%	0%	0%	0%	3.3%	6.7%	90.0%
CLOT	*C. albicans*	Vagina	21	≤2–64	95.2%	0%	0%	0%	0%	4.8%	0%
		Vulva	24	≤2	100.0%	0%	0%	0%	0%	0%	0%
	*N. glabratus*	Vagina	8	≤2–8	37.5%	12.5%	50.0%	0%	0%	0%	0%
		Vulva	7	≤2–8	57.1%	28.6%	14.3%	0%	0%	0%	0%
	*C. tropicalis*	Vagina	2	≤2	100.0%	0%	0%	0%	0%	0%	0%
		Vulva	2	≤2	100.0%	0%	0%	0%	0%	0%	0%
	*C. parapsilosis*	Vagina	2	≤2	100.0%	0%	0%	0%	0%	0%	0%
		Vulva	5	≤2	100.0%	0%	0%	0%	0%	0%	0%
	*S. cerevisiae*	Vagina	1	≤2	100.0%	0%	0%	0%	0%	0%	0%
		Vulva	0	-	0%	0%	0%	0%	0%	0%	0%
	*Rhodotorula* spp.	Vagina	0	-	0%	0%	0%	0%	0%	0%	0%
		Vulva	30	≤2–>64	70.0%	0%	6.7%	0%	10.0%	3.3%	10.0%

**Table 6:** The minimum inhibitory concentrations (MIC) to fluconazole (FLU) and clotrimazole (CLOT), as determined by broth microdilution, among the cases where isolates of the same species were isolated from both the vaginal and vulvar samples.

Species	ID	Clinical State	Niche	Fluconazole	Clotrimazole
*Nakaseomyces glabratus*	2	Vulvovaginal infection	Vagina	MIC ≤ 2	MIC ≤ 8
			Vulva	MIC ≤ 2	MIC ≤ 2
	8	Mild symptoms	Vagina	MIC > 64	MIC ≤ 8
			Vulva	MIC ≤ 64	MIC ≤ 2
		Mild symptoms	Vagina	MIC ≤ 64	MIC ≤ 2
			Vulva	MIC ≤ 32	MIC ≤ 2
		Mild symptoms	Vagina	MIC ≤ 16	MIC ≤ 4
			Vulva	MIC ≤ 32	MIC ≤ 4
		Mild symptoms	Vagina	MIC > 64	MIC ≤ 8
			Vulva	MIC ≤ 32	MIC ≤ 4
*Candida albicans*	35	Vulvovaginal infection	Vagina	MIC ≤ 64	MIC ≤ 2
			Vulva	MIC ≤ 8	MIC ≤ 2

## Data Availability

The data presented in this study is available within the article.
